# Clinical features and prognosis of immune checkpoint inhibitor-associated ocular inflammation in Japanese patients: a case series

**DOI:** 10.1007/s10384-025-01307-y

**Published:** 2025-11-22

**Authors:** Teruki Yamae, Yurika Aoyama, Hidetomo Izawa, Danni Shen, Hotaka Nemoto, Hiroshi Takase, Rie Tanaka

**Affiliations:** 1 Department of Ophthalmology, The University of Tokyo Hospital, Tokyo, Japan; 2 Department of Ophthalmology, Graduate School of Medicine, University of Tokyo, 7-3-1 Hongo, Bunkyo-ku, Tokyo, 113-8655 Japan

**Keywords:** Immune checkpoint inhibitors, immune–related adverse events, CTLA 4 inhibitors, PD–1 inhibitors, PD–L1 inhibitors

## Abstract

**Purpose:**

To investigate the clinical features of ocular inflammation caused by immune checkpoint inhibitors (ICIs) as immune-related adverse events (irAEs) in Japanese patients.

**Study design:**

Retrospective observational case series.

**Methods:**

We reviewed patients with ICI-associated ocular inflammation diagnosed at our institution in 2014–2024. Patient data—background characteristics, primary tumor, causative ICIs, time to onset, laterality, type of inflammation, irAE grade, treatment, visual outcomes, and complications—were analyzed. Stratified analyses were also performed according to the type of ICI.

**Results:**

The cohort included 7 males and 9 females (mean age, 62.1 ± 14.3 years). The most common primary tumor was malignant melanoma (8 patients). The causative ICIs were pembrolizumab, nivolumab, and nivolumab plus ipilimumab (5, 6, and 5 patients, respectively). The median time of onset was 64 days (12–952) post-administration, with significantly earlier onset in patients receiving nivolumab plus ipilimumab. Ocular inflammation was bilateral in 13 patients. Panuveitis (10 patients) was the most common inflammation type, and 6 patients exhibited Vogt–Koyanagi–Harada (VKH) disease–like uveitis. Grade 3 irAEs were the most frequent (11 patients). ICIs were discontinued in 15 patients and 9 required systemic corticosteroids. At the final follow-up, visual acuity had generally improved; however, 4 patients had a best corrected visual acuity of 20/40 or worse due to complications.

**Conclusion:**

In Japanese patients reviewed at our institution, ICI-associated ocular inflammation often presented as panuveitis, with VKH disease–like uveitis being relatively common. Visual impairment persisted in one-fourth of the patients, emphasizing the importance of careful management.

## Introduction

Immune checkpoint inhibitors (ICIs) are new cancer therapies that damage tumor cells by activating the body’s own immune system. Currently, there are eight ICIs approved for use in Japan: ipilimumab and tremelimumab (anti-CTLA-4 antibodies); nivolumab, pembrolizumab, and cemiplimab (anti-PD-1 antibodies); and durvalumab, atezolizumab, and avelumab (anti-PD-L1 antibodies). ICIs are used in monotherapy and in combination. Activation of the immune system can lead to adverse inflammatory events, termed immune-related adverse events (irAEs), which can occur in multiple organs. The incidence of irAEs is 64%–72% [1], and irAEs usually occur within 3–6 months after the start of ICI, although they may occur more than 1 year later [2, 3].

An ophthalmologic irAE is generally rare, with a reported incidence of 1%–2.8% [2, 4, 5]. Dry eye is the most common ophthalmologic irAE (1.2%–24.2%), followed by uveitis (0.3%–6%) [2]. Optic neuritis, myasthenia gravis, orbital myositis, corneal ulcer, hypotony, macular edema, and retinal vasculitis are also reported [2, 6, 7]. Melanoma (61.7%) is the most common malignancy reported as the primary cancer, followed by lung cancer (19.3%) and urological tumors (9.7%) [3]. Only a few cases of ICI-associated ocular inflammation have been reported from Japan, and there are no reports of large numbers of patients.

We investigated the clinical features of 16 Japanese patients with ICI-associated ocular inflammation diagnosed at our institution. To the best of our knowledge, this report represents the largest case series of ICI-associated ocular inflammation reported in Japan.

## Subjects and Methods

This retrospective study obtained approval from the ethics committee of the University of Tokyo (approval number: 2217) and conforms to the provisions of the Declaration of Helsinki in 1995 (as revised in Edinburgh, 2000). We used an opt-out consent process, wherein potential participants are informed about the study and their involvement, and participation is assumed unless they explicitly decline.

We investigated the clinical findings of consecutive patients with ICI-associated ocular inflammation who had visited the ophthalmology department of the University of Tokyo Hospital between January 2014 and September 2024. All patients were diagnosed with ICI-associated ocular inflammation after the exclusion of other potential causes of ocular inflammation based on ocular and systemic symptoms as well as laboratory tests.

The medical records were retrospectively reviewed to collect data on patient background, primary tumor, causative ICIs, tumor response to ICI therapy assessed using the revised RECIST guideline (version 1.1) [8], concurrent systemic irAE, time to onset, follow-up period, ocular symptoms, laterality, ocular inflammation type, anterior chamber cell grade at initial presentation (based on the Standardization of Uveitis Nomenclature [SUN] criteria) [9], best corrected visual acuity (BCVA), irAE grade (common terminology criteria for adverse events [CTCAE] Grading System [7, 10]), treatment, and ocular complications related to ocular irAEs. To incorporate visual acuity (VA) in the logarithmic system, we designated the VAs of counting fingers (CF) (50 cm), CF (5 cm), hand movements, light perception (LP), and no LP as 20/2000, 20/20 000, 20/40 000, 20/100 000, and 20/200 000, respectively [11]. BCVA was analyzed using logMAR values, and the mean logMAR was converted into Snellen equivalents for reporting.

All statistical data were analyzed using R version 4.3.1 (R studio Foundation for Statistical Computing). The demographic and clinical characteristics were compared between the three ICI groups: pembrolizumab, nivolumab, and nivolumab plus ipilimumab (nivolumab/ipilimumab). For statistical comparisons, we employed the Kruskal–Wallis test for continuous variables (age, time to onset, follow-up period, and CTCAE grade) and Fisher’s exact test for categorical variables (sex, ocular involvement laterality, malignant melanoma proportion, and VKH disease–like uveitis proportion). Whenever the Kruskal–Wallis test yielded statistically significant results, we conducted Dunn’s test with Bonferroni correction for post hoc pairwise comparisons. Similarly, for categorical variables with considerable results in Fisher’s exact test, pairwise Fisher’s exact tests with Bonferroni correction were conducted for the three-group comparisons. A *p*-value less than 0.05 was considered statistically significant.

## Results

### Demographics and Clinical Features of All 16 Cases

A total of 16 patients were identified with ICI-associated ocular inflammation. Table 1 shows the details for each patient. Table 2 summarizes the demographic characteristics of the 16 patients (7 [43.8%] males and 9 [56.3%] females, 62.1 ± 14.3 years old). The follow-up period was 13.6 ± 12.5 months (range, 1–53 months). The most common primary tumor was malignant melanoma (8 [50.0%] patients), followed by renal cancer (3 [18.8%] patients), and gynecological tumor, Hodgkin’s lymphoma, gastric cancer, esophageal cancer, and breast cancer (1 [6.3%] patient each). The causative agents were pembrolizumab in 5 (31.3%) patients, nivolumab in 6 (37.5%) patients, and nivolumab/ipilimumab in 5 (31.3%) patients. Systemic irAE were observed in 11 (68.8%) patients, with hepatopathy (4 [25.0%] patients) and dermatitis (3 [18.8%] patients) being the most common causes. The time from drug administration to the onset of ocular symptoms ranged from 12 to 952 days (median 64 days), and 10 (62.5%) patients had symptoms within 3 months of initiation. Subjective symptoms included blurred vision (8 [50.0%] patients), decreased VA (7 [43.8%] patients), and floaters (6 [37.5%] patients). Thirteen (81.3%) patients had bilateral and three (18.8%) patients had unilateral symptoms. The most common types of ocular inflammation were panuveitis (10 [62.5%] patients), followed by anterior uveitis (3 [18.8%] patients), and one (6.3%) patient each with sclerouveitis, posterior uveitis, and optic neuritis. Of the 15 (93.8%) patients with uveitis, 8 (50.0%) had granulomatous inflammation and 6 (37.5%) had Vogt–Koyanagi–Harada (VKH) disease–like uveitis. Among these six patients with VKH disease–like uveitis, only one (patient 15) exhibited an extraocular symptom, specifically vitiligo and poliosis. HLA genotyping was performed in two patients, and both were positive for HLA-DR4 (one for HLA-DRB1*04:05, and the other for DRB1*04:06). The severity of ocular irAE included 1 (6.3%) patient with Grade 1, 2 (12.5%) with Grade 2, 11 (68.8%) with Grade 3, and 2 (12.5%) with Grade 4.

**Table 1.** Patient characteristics

	Patient demographics						Ocular findings								Treatment & Complications		
	Age/sex Neoplasia	ICIs	Tumor response to ICI	Systemic irAE	Onset (days)	Follow-up (months)	Laterality	Inflammation type	Anterior chamber cell at initial visit	Granulomatous inflammation	VKH disease–like uveitis	Initial BCVA	Final BCVA	CTCAE Grade	ICI discontinuation reason	Ocular Treatment	Ocular complications related to ocular irAE
1	75/M Renal cancer	Pembro	SD	Diabetes Pancreatitis	15	5	Bilateral	Anterior uveitis	OD 1+ OS 1+		–	OD 0.7 OS 0.8	OD 1.0 OS 0.8	2	Temporary systemic irAE	Steroid eye drops	–
2	53/F Breast cancer	Pembro	N/A	Arthralgia	353	6	Unilateral	Anterior uveitis	OS 1+		–	OS 0.08	OS 0.8	3	Had already been completed as scheduled	Oral steroids	–
3	50/F Uterine cancer	Pembro	NE	Thyroiditis Dermatitis	73	8	Bilateral	Posterior uveitis	OD 0 OS 0		+	OD 1.2 OS 1.2	OD 1.2 OS 1.2	3	Permanent systemic irAE	Oral steroids	–
4	84/F Malignant melanoma	Pembro	PD		335	6	Bilateral	Panuveitis	OD 2+ OS 2+		+	OD 0.2 OS 0.5	OD 0.4 OS 0.6	3	Temporary ocular irAE	STTA	–
5	51/M Malignant melanoma	Pembro	CR		952	53	Bilateral	Panuveitis	OD 2+ OS 0.5+	+	–	OD 1.2 OS 0.7	OD 1.2 OS 1.0	3	Temporary ocular irAE	STTA	–
6	54/F Malignant melanoma	Nivo	PD	Hepatopathy	59	2	Bilateral	Anterior uveitis	OD 0 OS 0.5+	+	–	OD 1.0 OS 1.2	OD 1.2 OS 0.9	1	Permanent systemic irAE	Observation	–
7	40/M Hodgkin’s lymphoma	Nivo	Unknown	Dermatitis	228	10	Unilateral	Panuveitis	OD 0.5+		–	OD 0.4	OD 0.05	3	Permanent ocular irAE	Oral steroids	Hypotony maculopathy
8	67/M Renal cancer	Nivo	Unknown	Diarrhea	121	19	Bilateral	Panuveitis	OD 0.5+ OS 1+		–	OD 0.3 OS 0.4	OD 0.9 OS 0.7	3	Permanent ocular irAE	Steroid eye drops	–
9	65/M Renal cancer	Nivo	PR		67	2	Bilateral	Panuveitis	OD 1+ OS 0	+	+	OD 1.2 OS 1.2	OD 1.2 OS 1.2	3	Temporary ocular irAE	Steroid eye drops	–
10	80/F Gastric cancer	Nivo	SD		332	23	Bilateral	Panuveitis	OD 2+ OS 2+	+	+	OD 0.5 OS 0.5	OD 1.0 OS 1.2	3	Permanent ocular irAE	Oral steroids, STTA	–
11	70/M Esophageal cancer	Nivo	PD		40	1	Unilateral	Optic neuritis	OD 0		–	OD 0.004	OD 0.5	4	Permanent ocular irAE	Steroid pulse therapy	Optic atrophy
12	77/F Malignant melanoma	Nivo+Ipi	PD	Hepatopathy	12	10	Bilateral	Sclerouveitis	OD 1+ OS 1+	+	–	OD 0.5 OS 0.4	OD 0.3 OS 0.3	2	Temporary systemic irAE	Oral steroids	–
13	31/M Malignant melanoma	Nivo+Ipi	Unknown	Hypophysitis	61	25	Bilateral	Panuveitis	OD 0.5+ OS 0.5+		–	OD 0.8 OS 0.9	OD 0.6 OS 1.2	3	Permanent systemic irAE	Subconjunctival injection of dexamethasone	–
14	63/F Malignant melanoma	Nivo+Ipi	SD	Myocarditis Hepatopathy Dermatitis	24	16	Bilateral	Panuveitis	OD 1+ OS 1+	+	+	OD 1.2 OS 0.4	OD 1.2 OS 0.4	3	Permanent systemic irAE	Oral steroids	–
15	62/F Malignant melanoma	Nivo+Ipi	PR	Adrenal failure	13	16	Bilateral	Panuveitis	OD 1+ OS 2+	+	+	OD 0.8 OS 0.7	OD 0.4 OS 1.2	3	Temporary ocular irAE	Oral steroids STTA	CNV
16	72/F Malignant melanoma	Nivo+Ipi	SD	Hepatopathy Thyroiditis	12	15	Bilateral	Panuveitis	OD 1+ OS 1+	+	–	OD 0.09 OS 0.2	OD 0.1 OS 0.4	4	Permanent ocular irAE	Oral steroids	Suspected optic neuropathy

F, female; M, male; ICIs, immune checkpoint inhibitors; Pembro, pembrolizumab; Nivo, nivolumab; Ipi, ipilimumab; CR, complete response; PR, partial response; SD, stable disease; PD, progressive disease; N/A, not applicable, used in cases receiving adjuvant therapy without measurable disease; NE, not evaluable, not assessed because of early discontinuation before treatment response could be determined; VKH, Vogt–Koyanagi–Harada disease; BCVA, best corrected visual acuity (decimal scale); OD, right eye; OS, left eye; irAE, immune-related adverse event; CTCAE, common terminology criteria for adverse events; STTA, Sub-Tenon’s capsule triamcinolone acetonide injection; CNV, choroidal neovascularization

Anterior chamber cell grade was assessed using the Standardization of Uveitis Nomenclature (SUN) criteria

**Table 2.** Demographics

	anti-PD-1		anti-PD-1/anti-CTLA-4		
Checkpoint inhibitor	Pembrolizumab *n* = 5	Nivolumab *n* = 6	Nivolumab / Ipilimumab *n* = 5	*p-* value	Total *n* = 16
Patient demographics					
Age at presentation Mean ± SD (range)	62.6 ± 14.1 (50–84)	62.7 ± 12.7 (40–80)	61.0 ± 16.0 (31–77)	0.975^b^	62.1 ± 14.3 (31–84)
Female sex, n (%)	3 (60.0%)	2 (33.3%)	4 (80.0%)	0.388^a^	9 (56.3%)
Bilateral involvement, n (%)	4 (80.0%)	4 (66.7%)	5 (100.0%)	0.732^a^	13 (81.3%)
Time to onset (days) Median (range)	335 (15–952)	94 (40–332)	13 (12–61)	0.022^b^	64 (12–952)
Follow-up period (months) Mean ± SD (range)	15.6 ± 18.7 (5–53)	9.5 ± 8.7 (1–23)	16.4 ± 4.8 (10–25)	0.274^b^	13.6 ± 12.5 (1–53)
Primary cancer, n (%)					
Melanoma	2 (40.0%)	1 (16.7%)	5 (100.0%)	0.029^a^	8 (50.0%)
Renal cancer	1 (20.0%)	2 (33.3%)	0 (0.0%)		3 (18.8%)
Others	2 (40.0%)	3 (50.0%)	0 (0.0%)		5 (31.3%)
Type of ocular inflammation, n (%)					
Anterior uveitis	2 (40.0%)	1 (16.7%)	0 (0.0%)		3 (18.8%)
Posterior uveitis	1 (20.0%)	0 (0.0%)	0 (0.0%)		1 (6.3%)
Panuveitis	2 (40.0%)	4 (66.7%)	4 (80.0%)		10 (62.5%)
Sclerouveitis	0 (0.0%)	0 (0.0%)	1 (20.0%)		1 (6.3%)
Optic neuritis	0 (0.0%)	1 (16.7%)	0 (0.0%)		1 (6.3%)
VKH disease–like uveitis, n (%)	2 (40.0%)	2 (33.3%)	2 (40.0%)	1.000^a^	6 (37.5%)
CTCAE grade of ocular irAE, n (%)					
Grade 1	0 (0.0%)	1 (16.7%)	0 (0.0%)		1 (6.3%)
Grade 2	1 (20.0%)	0 (0.0%)	1 (20.0%)		2 (12.5%)
Grade 3	4 (80.0%)	4 (66.7%)	3 (60.0%)		11 (68.8%)
Grade 4	0 (0.0%)	1 (16.7%)	1 (20.0%)		2 (12.5%)

SD, standard deviation; VKH, Vogt–Koyanagi–Harada disease; irAE, immune-related adverse event; CTCAE, common terminology criteria for adverse events

Time to onset was the interval from immune checkpoint inhibitor administration to the ocular symptom onset.

n, the number of patients; no per-eye analysis was performed.

^a^Fisher’s exact test

^b^Kruskal–Wallis test

As shown in Table 1, ICIs were discontinued due to ocular symptoms (9 [56.3%] patients) or concurrent systemic irAE (6 [37.5%] patients). In one (6.3%) patient, treatment with ICI had already been completed as scheduled. Among the 15 patients who discontinued ICIs, 6 (40.0%) were temporary, while 9 (60.0%) were permanent. Six patients (37.5%) were treated with only topical corticosteroids, while 9 (56.3%) were treated with systemic corticosteroids (8 [50.0%] with oral steroids and 1 [6.3%] with steroid pulse therapy). One (6.3%) patient with anterior uveitis improved spontaneously with ICI discontinuation alone. Of the 9 (56.3%) patients who received systemic steroids, 6 (37.5%) were administered for ocular inflammation and 3 (18.8%) for concurrent systemic irAE.

The mean logMAR BCVA at the initial visit was 0.38 ± 0.62 in the right eye (OD) and 0.27 ± 0.32 in the left eye (OS). At the last observation (mean follow-up period, 13.6 ± 12.5 months; range, 1–53 months), the values were 0.25 ± 0.41 OD and 0.11 ± 0.20 OS. Excluding patients with pre-existing visual impairments such as cataracts, primary glaucoma, or amblyopia, four (25.0%) patients had BCVA of 20/40 or worse at the final observation due to ICI-associated ocular inflammation. The underlying causes were secondary choroidal neovascularization, hypotony maculopathy, optic neuritis, and suspected optic neuritis.

### Comparison between Pembrolizumab, Nivolumab, and Nivolumab/Ipilimumab Treatment Groups

Factors such as age (*p* = 0.975), sex (*p* = 0.388), laterality (*p* = 0.732), follow-up period (*p* = 0.274), CTCAE grade (*p* = 0.854), and the proportion of patients with VKH disease–like uveitis (*p* = 1.000) did not significantly differ between the three ICI groups (Table 2). Conversely, time to onset (*p* = 0.022) and the proportion of patients with malignant melanoma (*p* = 0.029) significantly differed between such groups. For the entire cohort, the median time to onset of ocular inflammation was 64 (12–952) days. When stratified by ICI type, it was 335 (15–952), 94 (40–332), and 13 (12–61) days in the pembrolizumab, nivolumab, and nivolumab/ipilimumab groups, respectively. The Kruskal–Wallis test demonstrated a statistically significant difference in the time to onset among the three ICI groups; thus, a post hoc analysis was conducted using Dunn’s test with Bonferroni correction. This analysis revealed that time to onset significantly differed between the pembrolizumab and nivolumab/ipilimumab groups (*p* = 0.023). The combination therapy of nivolumab and ipilimumab was associated with a short time to onset.

Among 16 patients, 8 (50.0%) had malignant melanoma, with 2 (40.0%), 1 (16.7%), and 5 (100.0%) in the pembrolizumab, nivolumab, and nivolumab/ipilimumab groups, respectively. Post hoc pairwise Fisher’s exact tests with Bonferroni correction revealed that melanoma prevalence significantly differed between the nivolumab/ipilimumab group and the nivolumab monotherapy group (adjusted *p* = 0.045).

## Discussion

Although the incidence of ophthalmic irAEs caused by ICIs is low (1%–2.8%) [2, 4, 5], reports of these AEs are increasing as the use of ICIs for malignancy becomes more widespread. Most reports remain limited to small case series and review articles, while in Japan, single case reports are even more predominant [12–25]. In this study, we retrospectively reviewed multiple aspects of these AEs at our single center. Such aspects included patient background, primary tumor, causative ICIs, concurrent systemic irAE, time to onset, ocular symptom, laterality, ocular inflammation type, BCVA, irAE grade, treatment, and ocular complications related to ocular irAE. Additionally, our study included a stratified analysis based on ICI type.

### Association with Different ICI Types

One retrospective cohort study reports that the causative agents for ocular irAEs were most common with the combination of anti-PD-1/anti-CTLA4 antibody therapy, followed by anti-CTLA-4 inhibitors, anti-PD-1 antibodies, and anti-PD-L1 antibodies, in that order [26]. In our current study, anti-PD-1 antibody therapy was given to 11 patients (nivolumab, *n* = 6; pembrolizumab, *n* = 5), and a combination of anti-PD-1/anti-CTLA-4 antibody therapy (nivolumab/ipilimumab) was given to 5 patients. These were approved in Japan earlier than other ICIs (nivolumab, 2014; pembrolizumab, 2015; ipilimumab, 2015; nivolumab/ipilimumab, 2018). Therefore, it is assumed that a relatively large number of patients have received anti-PD-1 antibody therapy in Japan. Previous reports [3, 26] also note that the rate of ocular irAE with anti-PD-L1 antibodies is low, and Kuo et al. [27] report that anti-PD-L1 antibodies do not increase the risk of developing ICI-associated uveitis. In Japan, anti-PD-L1 antibodies have been approved since 2017; however, no cases of ocular inflammation associated with anti-PD-L1 antibodies were identified in our study.

Melanoma was the most common primary cancer in our cohort, accounting for 50% of cases, consistent with previous reports [2]. In Table 2, the number of patients with melanoma as the primary disease was relatively low in the nivolumab monotherapy group. This result may be attributed to the fact that nivolumab was the first ICI to be approved in Japan, with its indications having been expanded to various malignancies.

By comparing the pembrolizumab, nivolumab, and nivolumab/ipilimumab groups, the nivolumab/ipilimumab group had the shortest time from drug initiation to ocular inflammation onset. Similarly, Sun et al. report that the time between drug initiation and uveitis onset was shorter in patients treated with ipilimumab than in those treated with pembrolizumab or nivolumab monotherapy, with all cases occurring within 20 days [28].

### Characteristics of Ocular Inflammation

In our current study, granulomatous inflammation was observed in 8 of 15 (53.3%) patients with uveitis, consistent with a previous report documenting granulomatous inflammation in 60% of ICI-associated uveitis cases [29]. Dow et al. conducted a comprehensive literature review and report that in 241 eyes of 126 patients with ICI-associated uveitis, anterior uveitis was the most common (37.7%), followed by panuveitis (34.0%), posterior uveitis (25.7%), and intermediate uveitis (1%) [10]. This distribution was similar regardless of the ICI used, but patients receiving a combination of ICIs demonstrated a higher rate of anterior uveitis [10]. According to several review articles, 8.3%–11.9% of patients with ICI-associated uveitis presented with VKH disease–like uveitis [3, 10, 30]. Zhang et al. conducted a literature review and analyzed cases of 41 patients with VKH disease–like uveitis associated with ICIs [2]. The majority of patients were women with a mean age of 68 years, most of whom (59%) were being treated for melanoma with a PD-1 or PD-L1 inhibitor (76%). In our study, among the 15 patients with uveitis, panuveitis was the most common type (n = 10, 66.7%), followed by anterior uveitis (n = 3, 20%). Among the 15 patients, 6 presented with VKH disease–like uveitis. Of these 6, 5 were women (83.3%), the primary cancer was melanoma in 3 (50%), and all patients had been treated with anti-PD-1 antibodies. Another report of 9 patients from Japan, found panuveitis in 7 (77.8%) and posterior uveitis in 2 (22.2%) patients. They also report that VKH disease–like uveitis was present in 5 of 9 patients (55.6%) [25]. Although Takeuchi et al.’s report and our current study involve a small number of patients, they suggest that panuveitis is more commonly observed in Japanese patients, with a potentially higher prevalence of VKH disease–like uveitis in this population. Case reports describe links between ICI-associated VKH disease–like uveitis and HLA-DRB1*04:05 [22–25]. Among the six patients with VKH disease–like uveitis at our hospital, HLA testing was performed in two, with one testing positive for HLA-DRB1*04:05. The frequency of HLA-DRB1*04:05 is particularly high in patients with VKH, with a reported relative risk of 9.5 [31]. Given the high frequency of HLA-DRB1*04:05 in the Japanese population, VKH disease–like uveitis is likely to be more common in this population.

Reports of ICI-associated optic neuritis are rarer than those of uveitis. Francis, et al. report that ICI-associated optic neuritis tends to be bilateral, characterized by painless visual decline, and typically occurs within four cycles of ICI therapy [32]. They also note that visual function stabilized following drug cessation and systemic steroid administration. In our current study, we observed one case of unilateral optic neuritis that developed after three cycles of nivolumab. At the initial visit, the patient’s BCVA was finger counting; however, after three courses of steroid pulse therapy, it improved to 20/40.

### Management of Ocular Inflammation Associated with ICIs

Treatment recommendations based on the CTCAE grading scale have been published by the American Society of Clinical Oncology, the Society for Immunotherapy of Cancer, and the National Comprehensive Cancer Network [2]. According to these recommendations, the decision to discontinue ICIs may be considered based on the severity of ocular inflammation. Discontinuation of ICIs is recommended for uveitis classified as Grade 2 or higher, with re-initiation advised only after the inflammation improves to Grade 1 or lower. In our study, 15 patients had ICIs discontinued due to ocular irAEs or systemic irAEs. Of these 15, 9 were due to ocular inflammation (7 with Grade 3 and 2 with Grade 4). Chaudot et al. demonstrate an association between the severity of uveitis and the incidence of ICI discontinuation. They found that ICIs were stopped in 45% of anterior uveitis cases, 50% of intermediate uveitis, 33% of posterior uveitis, and 71% of panuveitis cases [29]. In another review, ICIs were discontinued in 51.4% of patients and suspended in 11.4% [10]. Although the CTCAE grading system provides a standardized framework for evaluating adverse events, it may not completely reflect clinical reality. Thus, ICI discontinuation should be carefully considered according to ocular inflammation severity, primary cancer status, systemic irAEs, and treatment alternatives. Such decisions are best made through a careful discussion among oncologists, ophthalmologists, and other relevant specialists.

The CTCAE guidelines recommend the use of local corticosteroid therapy for low-grade uveitis, local and/or systemic steroids for moderate uveitis (CTCAE grade 2), and high-dose systemic steroids for more severe uveitis (CTCAE grades 3–4). Administration of systemic steroids raises concerns about the suppression of immune responses, as well as the risk of causing deterioration of the overall condition of cancer patients. In a review, Chaudot et al. found that 58% of anterior uveitis patients had been treated with local steroids only [29]. Additionally, it is reported that among 26 patients with panuveitis, 42% were treated with local steroids alone, 35% with a combination of local and systemic steroids, and 19% with systemic steroids alone [29]. Takeuchi M et al. report nine cases of ICI-associated uveitis with posterior segment involvement. Among these, only two patients required systemic steroid administration, while six were treated with periocular corticosteroid injections, and one with steroid eye drops alone [25]. Similarly, in our current study, of the 11 patients with Grade 3 ocular irAEs with posterior segment inflammation, 5 were treated with local steroids (two with steroid eye drops, one with subconjunctival dexamethasone injection, and two with sub-Tenon’s triamcinolone acetonide injections). Even in cases of ICI-associated uveitis with posterior segment involvement, local steroid therapy may be considered depending on the severity of ocular inflammation and the degree of visual impairment.

### Visual Prognosis and Ocular Complications

In patients with ICI-associated ocular inflammation, the degree of visual impairment substantially varies. In a review of 126 patients with ICI-associated uveitis, the median initial VA during diagnosis was 20/40. The median final VA improved to 20/25, with 68.5% of patients achieving 20/30 or better vision, whereas 11.5% had a final VA of 20/200 or worse [10]. Herein, the mean BCVA at the initial visit was 20/43. At the last observation, the mean BCVA was 20/31. Four patients (25%) had a BCVA of 20/40 or worse at the final observation due to ICI-associated ocular inflammation. The underlying causes were secondary choroidal neovascularization, hypotony maculopathy, optic neuritis, and suspected optic neuritis. Among them, the patient with choroidal neovascularization (case 15) was being treated with intravitreal antivascular endothelial growth factor injections; however, visual improvement had been limited. Moreover, the patient with optic neuritis (case 11) and the patient with suspected optic neuritis (case 16) underwent systemic corticosteroid therapy; however, visual function remained poor. Hypotony maculopathy (case 7) is considered difficult to treat. Decreased BCVA is reportedly caused by hypotony maculopathy [33], epiretinal membrane [34], macular edema [34], choroidal neovascularization [35], and optic nerve pallor [36]. As previously noted, panuveitis is predominantly reported in Japanese patients; therefore, attention should be paid to the development of this vision-threatening complication.

Our study has several limitations. First, it is a single-center, retrospective study with a small sample size. Further research with a larger sample size is warranted to clarify the characteristics of ICI-associated ocular inflammation in Japan. Second, as our institution is a university hospital that frequently receives referrals of severe cases, it is possible that there were fewer patients with mild ocular irAEs. Third, in some patients, the follow-up period was short, and the long-term visual prognosis remained unclear. Despite these limitations, we believe this study provides valuable insights into the characteristics of ICI-associated ocular inflammation in the Japanese population.

In conclusion, our retrospective review of 16 Japanese patients with ICI-associated ocular inflammation demonstrates that panuveitis was a frequent manifestation, with VKH disease–like uveitis being relatively common. Ocular inflammation tended to occur earlier in the nivolumab/ipilimumab group than in the pembrolizumab group. Although most patients responded to treatment, visual impairment persisted in one-fourth of the patients, highlighting the importance of early recognition and careful management of ocular irAEs.
